# Surface waves control bacterial attachment and formation of biofilms in thin layers

**DOI:** 10.1126/sciadv.aaz9386

**Published:** 2020-05-27

**Authors:** Sung-Ha Hong, Jean-Baptiste Gorce, Horst Punzmann, Nicolas Francois, Michael Shats, Hua Xia

**Affiliations:** Research School of Physics, The Australian National University, Canberra, ACT 2601, Australia.

## Abstract

Formation of bacterial biofilms on solid surfaces within a fluid starts when bacteria attach to the substrate. Understanding environmental factors affecting the attachment and the early stages of the biofilm development will help develop methods of controlling the biofilm growth. Here, we show that biofilm formation is strongly affected by the flows in thin layers of bacterial suspensions controlled by surface waves. Deterministic wave patterns promote the growth of patterned biofilms, while wave-driven turbulent motion discourages patterned attachment of bacteria. Strong biofilms form under the wave antinodes, while inactive bacteria and passive particles settle under nodal points. By controlling the wavelength, its amplitude, and horizontal mobility of the wave patterns, one can shape the biofilm and either enhance the growth or discourage the formation of the biofilm. The results suggest that the deterministic wave-driven transport channels, rather than hydrodynamic forces acting on microorganisms, determine the preferred location for the bacterial attachment.

## Teaser

Wave-controlled transport in fluid provides a novel method to shape and promote biofilm formation for bioengineering applications.

## INTRODUCTION

Recent progress in understanding fluid motion driven by surface waves allows better prediction of transport of matter and the development of new tools to manipulate particles in fluids. This is important for many applications ranging from spreading of the pollutants to clustering and settlement of living microorganisms. Faraday waves, or parametrically excited surface waves (*1*), have been well characterized and understood because of a large body of laboratory experiments and numerical simulations. At low excitation amplitude, these nonlinearly generated waves form stable patterns (*2*–*5*), and as the forcing is increased, the patterns become unstable, eventually creating turbulent motion of fluid in the horizontal plane (*6*, *7*). Such turbulence reproduces, in detail, the statistics expected in ideal two-dimensional (2D) turbulence (*8*–*12*). Disordered Faraday waves can be described as ensembles of oscillating solitons, also referred to as oscillons (*13*–*15*). The oscillon description of such disordered wave fields allows us to characterize transitions from linear regime to turbulence via the introduction of horizontal mobility of oscillons performing random walk. Such oscillon motion determines the diffusive transport of fluid particles on the surface (*16*). Because waves also generate fluid motion below free surfaces, such motion should also affect transport of particles and sedimentation near the solid bottom in thin fluid layers. As we show below, the wave-driven transport is essential for the process of settlement and attachment of microorganisms, such as bacteria, during early stages of the formation of biofilms.

Although many bacterial species can self-propel, they colonize submerged surfaces, forming biofilms, the most abundant form of bacterial life (*17*). Biofilms are the self-produced aggregates of microorganisms in which bacteria are embedded in a complex 3D matrix of extracellular polymeric substances. While bacterial biofilms are often associated with their adverse effects on surrounding organisms or hosts (medical implant surfaces, water pipes, etc.), many bacteria are beneficial for a variety of environmental, engineering, and medical applications such as water treatment and new material technologies (*18*–*20*). Many applications can benefit from the ability to control and shape the growth of biofilms, for example, for the development of bacterial scaffolds for tissue engineering (*21*, *22*) or for growing bacterial cellulose, a structural component of some biofilms (*23*).

The microbial consortia can be shaped using different patterning techniques. Biofilm lithography has been used to pattern biofilms using optically controlled microbial gene expression. Patterned substrate modification, together with specific antibodies, was used to immobilize and pattern live bacterial cells (*24*). Communities of different bacterial species have been constructed using microfluidic devices to control spatial structure and chemical communication (*25*). Bacteria are also sensitive to physical stimuli and mechanical cues such as hydrodynamic forces, adhesive forces, and the rheology of their surroundings (*26*–*28*). For example, it was shown recently that fluid flows control the microscopic structure and 3D morphology of biofilms (*29*). Other mechanical factors, such as mechanical vibration of the substrate, also affect bacterial attachment, leaving a footprint on the biofilms (*30*).

The motion of a liquid driven by surface waves must play important roles in the process of bacterial attachment to the solid substrate and on the biofilm development in shallow layers. On one hand, wave-driven hydrodynamic forces may encourage the settlement of bacteria at particular spots under waves. On the other hand, the wave-driven flows may create favorable conditions for the biofilm development by establishing the delivery of nutrients, oxygen, or other essential components to the biofilm location. Recent progress in understanding the motion of particles in fluids whose surfaces are perturbed by hydrodynamic waves offers new ideas on how such flows can be used to control and shape the formation of biofilms.

Here, we report the development of bacterial biofilms at the bottom of vertically vibrated containers in thin layer of bacterial suspensions. To uncover factors affecting bacterial attachment, we image surface waves, visualize sedimentation of passive microparticles and inactive bacteria, image vertical mixing of a passive scalar, and study biofilms developing at the bottom of the fluid container.

We find that structured, deterministic wave-driven flows encourage the development of strong biofilms attached to the bottom of the microplate wells. Such biofilms are characterized by regular periodic patterns, which are well correlated with the patterns of the surface waves. Biofilms are formed under the wave antinodes (the peak-trough locations), while inactive bacteria and passive particles accumulate under nodal points where the surface elevation is constant in time. The biofilm patterns are scalable: The characteristic spatial period of a pattern can be adjusted by changing the frequency of the vertical vibration and its amplitude. At higher wave amplitudes, the wave field becomes disordered, leading to turbulent horizontal transport and intense mixing in the fluid. In this regime, biofilms do not develop.

## RESULTS

### Biofilm formation under surface waves

Surface waves induce motion of fluid particles on the surface of a liquid and in the bulk. For linear, small amplitude waves, the velocities of fluid particles exponentially decrease as a function of the distance from the surface. The depth of the liquid is an important factor affecting both the wave dispersion relation and trajectories of particles settling at the bottom and, thus, the sedimentation efficiency (*2*, *31*, *32*). The dispersion relation of waves in the finite-depth fluid layer is given by $\omega^{2}=(\mathrm{gk}+\frac{\sigma }{\rho }k^{3})\text{tanh}(\mathrm{kh})$, where ω is the wave frequency, *h* is the layer depth, *k* = 2π/λ is the wave number, σ is the surface tension coefficient, *g* is the acceleration of gravity, and ρ is the density of the liquid. If a fluid container is vertically vibrated at the frequency *f*_s_ with sinusoidal acceleration above a certain threshold, then Faraday waves at the frequency of *f*_F_ = *f*_s_/2 are excited at the liquid surface. Here, we investigate the biofilm formation in the presence of Faraday waves excited at *f*_s_ from 45 to 120 Hz in a broad range of vertical accelerations. Detailed studies are performed at *f*_s_ = 120 Hz (*f*_F_ = 60 Hz), which corresponds to the Faraday wavelength of λ_F_ ~ 5 mm. The layer thickness of the solution is kept at *h* = 2 mm for most experiments to allow waves to affect the motion of the fluid at the bottom, satisfying a shallow water layer condition (*h*/λ_F_ < 0.5). Thicker layers of fluids do not show strong patterning of biofilms.

**Fig. 1:**
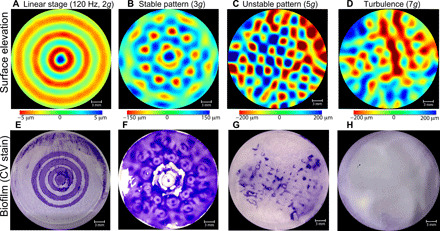
Patterns of waves and biofilms produced by *E. coli* (ATCC 25966) bacteria at different accelerations. (**A** to **D**) Contour plots of the measured instantaneous surface elevation. Red and blue colors correspond to peaks/troughs of the waves. (**E** to **H**) Corresponding images of the CV stain of the biofilm attached to the bottom of the microplate after exposure of the bacterial suspension to the waves for 24 hours. Darker colors correspond to thicker biofilms. Microplates are vibrated at *f*_s_ = 120 Hz at the vertical accelerations of (left to right) *a* = 2*g*, 3*g*, 5*g*, and 7*g*.

Figure 1 shows the results from four experiments in which a well of a microplate filled with the bacterial suspension of *Escherichia coli* is vertically vibrated at the frequency of 120 Hz at different vertical accelerations *a*. The wave patterns, measured using synthetic Schlieren technique (*33*) (see Materials and Methods), show circular wavefronts for standing waves at *a* = 2*g*, stable flower-shaped pattern at *a* = 3*g*, unstable but ordered square pattern at *a* = 5*g*, and a constantly evolving in time turbulent wave field at *a* = 7*g*. Biofilms that develop during 24 hours of exposure to such waves are illustrated in the bottom row. The biofilms [visualized using crystal violet (CV) stain] have the spatial structure similar to that of the Faraday waves at lower accelerations of *a* = 2 to 5*g*, while in the turbulent wave field at *a* = 7*g*, no biofilm pattern is observed. The number of rings in the biofilm at *a* = 2*g* is twice the number of the wave periods of the wave.

**Fig. 2:**
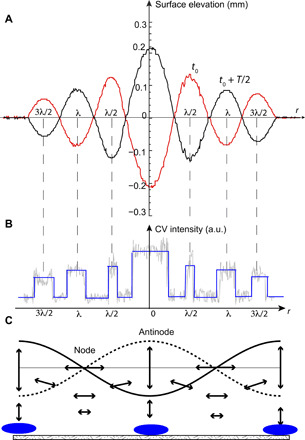
Location of biofilms at the vertical acceleration of 2*g*. (**A**) Profiles of the surface elevation produced by the Faraday wave (*a* = 2*g*) at two time instants *t*_0_ and *t*_0_ = *t* + *T*/2 (where *T* is the wave period), and (**B**) corresponding profile of a CV intensity (approximately proportional to the biofilm thickness). a.u., arbitrary units. (**C**) Schematic of the fluid motion under a standing surface wave (*41*). Blue areas at the bottom indicate the locations of the biofilm growth.

The comparison between the biofilm strength (derived from the CV intensity) and the amplitude of the surface elevation (Fig. 2, A and B) shows that thicker biofilms form under the locations of the wave antinodes. As mentioned above, the number of rings in the biofilm at *a* = 2*g* is twice the number of the wave periods of the wave (Fig. 1). The biofilm pattern corresponds to two antinodes per wave period. The fluid motion at the antinodes in a standing wave is vertical, while at the positions of the wave nodes, fluid particles move almost horizontally, as illustrated in the schematic of Fig. 2C. As discussed below, the position of the biofilm under the standing waves does not coincide with the expected locations of the sedimentation of the passive microparticles. Generally, a higher wave amplitude at a particular location leads to a thicker biofilm underneath it, as seen in Fig. 2 (A and B).

**Fig. 3:**
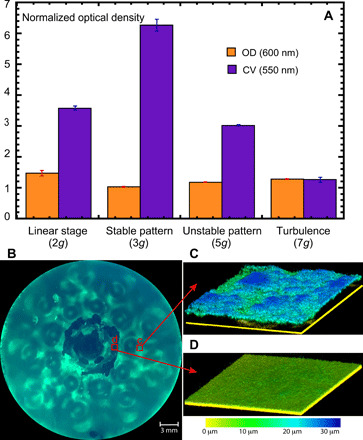
Biofilm production. The bacterial suspensions are vibrated for 24 hours and then incubated for another 24 hours at 37°C. (**A**) Total mass of the biofilms (measured as the normalized absorption or the OD of the CV stain at 550 nm) generated in a microplate well (35 mm diameter) at four different vertical accelerations (the vibration frequency is 120 Hz) (violet bars). The OD of the bacterial suspension (measured at 600 nm) is about the same in all experiments (orange bars). Measurements are performed six times with three repeats each at every vertical acceleration. The OD is normalized by the OD of the control (no-shaken) samples. The error bars show the SD of the measurements. (**B**) Fluorescent image of the biofilm formed in a well exposed to vibration at the vertical acceleration of 3*g*. Lighter colors correspond to thicker biofilm. (**C** and **D**) Reconstructed 3D surface of the biofilm using the CLSM at two regions of interest (200 × 200 μm) indicated by the red boxes in (B).

The total mass of the biofilm within a microplate depends not only on the wave amplitude but also on the stability of the wave pattern (Fig. 3A). The biomass is estimated from the measurements of the optical density (OD) of the CV stain in the biofilm measured at the wavelength of 550 nm. The light absorption in the CV solution (OD_550_) is compared with the control sample to obtain the normalized biofilm growth. Similarly, the OD at the wavelength of 600 nm is measured and compared with the OD of the control sample to evaluate the planktonic bacteria growth. For the approximately constant density of the bacterial suspension, the OD of the CV is noticeably (three to six times) higher in the samples exposed to the surface waves in comparison with the control (nonvibrated) samples. The mass of the biofilm has a maximum at the vertical acceleration of *a* = 3*g*. This corresponds to a reasonably intense wave still showing stable patterns. At higher acceleration, in the presence of moving unstable patterns (*a* = 5*g*), the mass is decreased. For a turbulent wave field at *a* = 7*g*, the biofilm is not formed, same as is in the control sample. In other words, the biofilm development is the strongest in the presence of stationary wave patterns, while the increase in the horizontal mobility of the wave crests reduces the biomass and does not encourage the pattern formation (see below).

Measurements of the biofilm thickness are also performed using the confocal laser scanning microscopy (CLSM). Figure 3B shows the fluorescent image of the thick biofilm developed at *a* = 3*g*. CLSM images of two regions of interest indicate that within the thicker region, the biofilm thickness is about 20 to 25 μm (Fig. 3C), while at the minimum, it is a monolayer of microorganisms (Fig. 3D), similar to the control sample: 2 to 4 μm.

### Wave amplitude and horizontal mobility determine the biofilm growth

Parametrically excited waves in vertically vibrated containers become disordered at higher vertical accelerations (*6*, *7*, *14*). A wave in a circular well develops at the first subharmonic of the vibration frequency *f*_F_ = *f*_s_/2 above some acceleration threshold. At higher acceleration, the regular wave structure (concentric rings) is modulated by the cross-wave instability until the wave is broken into a wave field consisting of individual oscillating solitons, or oscillons (*34*). This is illustrated in fig. S1, which shows the first few seconds of the development of the parametrically excited wave.

**Fig. 4:**
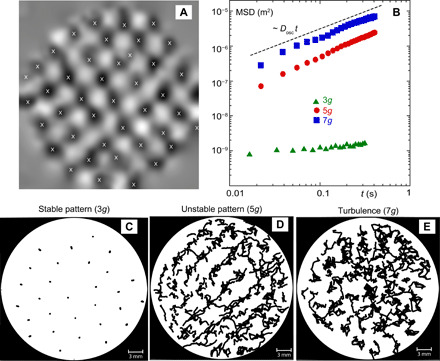
Oscillon statistics. (**A**) Illustration of the identification of the oscillons as the local maxima in the measured wave field at *5g*. (**B**) MSD of the oscillons at different vertical accelerations. (**C** to **E**) Trajectories of the oscillons tracked over 0.4 s (24 wave periods) at different vertical accelerations (left to right): 3*g*, 5*g*, and *7g.*

With the increase in the acceleration, the peak-to-peak height of the wave increases from ~0.01 mm at 2*g* to ~0.4 mm at 7*g*. The motion of individual oscillons becomes random: They chaotically move, collide, and merge (see movie S1). Such wave fields can be analyzed by viewing oscillons as quasiparticles. The oscillons are identified as the local maxima in the wave field, marked in Fig. 4A by the crosses. The diffusion coefficient characterizing the random walk motion of the oscillons [derived from the mean-squared displacement (MSD) of the maxima of the local surface elevation] is directly related to the fluid particle dispersion at the fluid surface (*16*). In a stable wave pattern, such as the one in Fig. 1B, oscillons move very slowly around their equilibrium positions, but as the vertical acceleration is increased (Fig. 1, C and D), the horizontal motion of oscillons becomes essential for the wave dynamics. Figure 4 (C to E) shows trajectories of oscillons in the horizontal plane for three vertical accelerations corresponding to the wave fields shown in Fig. 1 (B to D). Figure 4B shows the MSD of oscillons as a function of time 〈δ*r*^2^〉 = 〈(*r*(*t*) − *r*(0))^2^〉. At long times, the MSD is proportional to time. This indicates the diffusive nature of the process 〈δ*r*^2^〉 = 2*D*_osc_*t*. At low vertical acceleration (*a* = 3*g*), corresponding to the case of the stable pattern of Fig. 1B, the MSD is small, while at higher accelerations, it is increased by up to four orders of magnitude (Fig. 4B).

The diffusion coefficient *D*_osc_ is a measure of the oscillon horizontal mobility, and it is proportional to their root mean square velocity ${\langle {\tilde{u}}_{\text{osc}}\rangle }_{\text{rms}}$. This velocity is linearly proportional to the rms velocity of the fluid particles at the fluid surface (*16*) ${\langle \tilde{u}\rangle }_{\text{rms}}\approx 3{\langle {\tilde{u}}_{\text{osc}}\rangle }_{\text{rms}}$. In these experiments, the fluid thickness is less than the wavelength (*h*/λ ~ 0.4) such that the fluid motion at the free surface also affects the motion near the bottom of the fluid well. We perform measurements of motion of passive particles at the bottom to compare with that of the active bacteria.

**Fig. 5:**
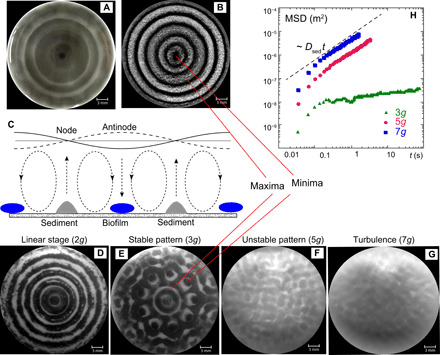
Sedimentation of passive particles and bacteria. (**A**) Image of the sedimentation of inactive *E. coli* bacteria at the vertical acceleration of *2g*. (**B**) Image of the sedimentation of polyamid particles (0.005 mm diameter, specific density of 1.03) at the vertical acceleration of *2g.* (**C**) Schematic of the streaming pattern under the standing surface waves (*32*). Blue and gray areas at the bottom indicate the locations of the biofilm growth and of the sedimentation of passive particles, respectively. (**D** to **G**) Sedimentation pattern of the titanium dioxide powder (0.0004 mm diameter, specific density of 3.0) at different vertical accelerations: 2*g*, 3*g*, 5*g*, and 7*g*. (**H**) Corresponding MSD of the sedimentation patterns at different vertical accelerations.

The sedimentation of the inactive bacteria in phosphate-buffered saline with no nutrients is shown in Fig. 5A (see Materials and Methods for details). The bacterial solution is subject to vertical oscillations at the frequency of 120 Hz and acceleration of 2*g* for 4 hours. The sedimented bacteria at the bottom of the microplate are seen as white circular rings. No bacteria settlement is observed under the antinode in the center of the microplate well. The positions of the rings correspond to the nodal points of the surface waves on the free surface.

We compare patterns of the inactive bacteria with the sedimentation patterns of synthetic passive microparticles: titanium dioxide (white, 0.0004 mm diameter, specific density of 3) and polyamid seeding particles (white, 0.005 mm diameter, specific density of 1.03) at the vertical acceleration of 2*g*. The sedimentation patterns of these microparticles are shown in Fig. 5 (B and D). The locations of maxima and minima of synthetic particles are similar to those of inactive bacteria (Fig. 5A). The maxima/minima locations are out of phase when compared with the biofilm pattern shown in Fig. 1E.

Figure 5 (D to G) shows patterns of the powder of titanium dioxide sedimented at different accelerations at the bottom of the microplate wells. These patterns are similar to the biofilm patterns (Fig. 1, E to H). The main difference is that the passive particles are accumulated under the wave nodes, while the maxima of the biofilms correspond to antinodes (Fig. 5C).

The sedimentation of passive particles under waves is believed to be due to a wave streaming effect, or the generation of the time-averaged motion due to the rectification of the fluctuations of the fluid velocity (for example, due to the Reynolds stress). The streaming motion was first considered by Rayleigh for acoustic waves (*35*, *36*), and it was later termed “steady streaming” in incompressible flows (*37*). Figure 2C illustrates the fast oscillating fluid motion induced by the wave. In addition to these oscillations occurring at the wave frequency, the surface wave induces a slow steady streaming motion shown in Fig. 5C. The streaming moves fluid down from the antinodes and up under the nodal points. Such a streaming pattern has been confirmed experimentally in the Faraday waves (*32*). In our experiments, the sedimentation of passive particles is also observed at the nodal points, in agreement with previous observations (*31*) and consistent with the streaming motion.

The increased horizontal mobility of the surface waves leaves a footprint on the sedimentation patterns. These patterns become blurry at higher accelerations *a* = 5 to 7*g*. We track the sedimented particles near the bottom of the well. The procedure is similar to the tracking of the oscillons shown in Fig. 4A. The local maxima in the sedimentation patterns are identified and then tracked for up to 1 hour. The MSD of the sediment as a function of time is shown in Fig. 5H for the vertical acceleration in the range of *a* = 3 to 7g. The MSD reveals the diffusive nature of the sediment motion 〈δ*r*^2^〉 = 2*D*_sed_*t*. The MSD is rather low in the presence of the stable wave pattern (*a* = 3*g*), while in the presence of the unstable pattern (*a* = 5*g*) and in turbulence (*a* = 7*g*), the diffusion coefficient *D*_sed_ is higher by a few orders of magnitude. It was shown in (*11*) that on the surface of the fluid perturbed by steep Faraday waves, the horizontal diffusion coefficient is given by $D={\langle \tilde{u}\rangle }_{\text{rms}}L_{f}$, where *L*_f_ = λ/2 is the forcing scale of the surface flow, which is approximately equal to half the Faraday wavelength. In our experiments *L*_f_ ~ 2.5 mm at the frequency of the parametrically excited waves of *f*_F_ = 60 Hz. From the diffusion coefficient *D*_sed_ of the sedimented particles of Fig. 5F, we estimate the rms particle velocity at the bottom ${\langle {\tilde{u}}_{\text{sed}}\rangle }_{\text{rms}}$ to be about 0.01 mm/s for the case of a stable pattern (*a* = 3*g*) and about 1 mm/s in the turbulent regime at *a* = 7*g*. The former velocity (0.01 mm/s) is less than the motility of active *E. coli* bacteria, which is in the range of 0.015 to 0.07 mm/s (*38*). Thus, the microorganisms can overcome the wave-induced fluid motion at lower wave amplitudes (<3*g*), while in the turbulent regime, the wave-driven flow dominates over their motility.

## DISCUSSION

Faraday waves in thin layers of media generate flows that affect the attachment of bacteria to the microplate bottom and the overall growth of biofilms. The patterns of mature biofilms (24 hours of shaking plus 24 hours of incubation) reproduce patterns of the surface waves. The maximum thickness of the biofilms is correlated with the locations of the peaks-troughs of the waves. The settlement of passive microparticles occurs under the wave nodal points, where the surface elevation does not change in time.

The sedimentation patterns of the passive microparticles are in agreement with the expected streaming patterns (*35*–*37*) and also with recent experimental observations (*31*, *32*). To exclude the possibility that the size and the density of the sedimented synthetic microparticles play a role in their settlement locations, we performed experiments with inactive *E. coli* bacteria in the suspensions of phosphate-buffered saline with no nutrient. In such solutions, bacteria show substantially reduced motility (*39*) and thus should sediment similarly to passive microparticles. The settlement of inactive microorganisms is observed at the wave nodes (Fig. 5A). This suggests that the ability of active *E. coli* to overcome the wave-produced flows is an important factor that determines the selection of the attachment location at the bottom. When the fluid velocities exceed the swimming speed of the bacteria, no biofilm patterns are observed.

Among other factors which determine the location of the biofilm, can be the wave-driven transport routes, delivering, for example, oxygen. It has been found that in the nutrient broth, *E. coli* cells use oxygen very quickly, and once oxygen is exhausted, the average velocity of the bacteria markedly decreases (*40*). It is thus possible that the transport routes connecting the fluid surface with the bottom of the container, due to the wave streaming, is the main factor determining the locations of the biofilm development.

To test how the streaming transport is affected by the horizontal mobility of the waves, we visualize mixing of the passive scalar (a fluorescent dye initially placed at the fluid surface) into the bulk (see fig. S2). In the presence of stable wave patterns at *a* = 2 to 3*g*, the mixing occurs in the form of well-defined stationary vertical plumes penetrating from the antinodes at the free surface to the bottom of the well. At higher accelerations, intense horizontal mixing of the fluid destroys vertical plumes, thus destroying deterministic transport routes from the fluid surface.

**Fig. 6:**
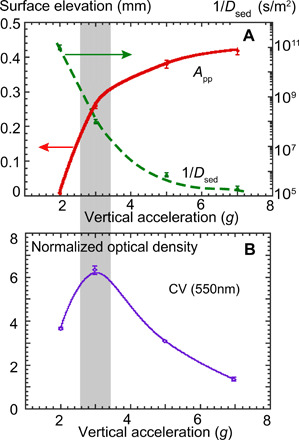
Biofilm formation and wave parameters. (**A**) Amplitude of the surface elevation (red solid line) and the inverse of the diffusion coefficient of the oscillons (green dashed line), and (**B**) the total mass of attached biofilm, as a function of the vertical acceleration.

The maximum production of the biofilm mass is observed at the intermediate wave intensity and at vertical accelerations corresponding to stable wave patterns and low horizontal transport of a fluid. This is illustrated in Fig. 6, summarizing data on the biofilm production, the wave height, and the wave mobility. Figure 6A shows the peak-to-peak wave amplitude of the surface elevation and the inverse diffusion coefficient of the sedimented particles (1/*D*_sed_) as a function of vertical acceleration. The total mass of the biofilms, estimated from the OD of the CV stain at 550 nm, is shown in Fig. 6B. The strongest biofilm (shaded region) is observed at a finite wave height and relatively low horizontal mobility (∝*D*_sed_) of the microparticles near the bottom. In the fully turbulent flow at *a* = 7*g*, no patterns are observed, and the biofilm mass is the lowest.

The above results are scalable. By changing the frequency of the vertical vibration in the range of *f*_s_ = 45 to 120 Hz (and the Faraday wavelength), we observe different spatial scales of the wave-induced patterns of biofilms (see fig. S3). The lower the *f*_s_, the larger the scale of the biofilm patterns.

The wavelength is a parameter that determines the thickness of the fluid layer in which biofilms are affected by the surface waves. We compare the biofilm growth at *f*_s_ = 45 Hz (λ ~ 10 mm) with the above measurements at *f*_s_ = 120 Hz (λ ~ 5 mm). Figure S4 shows the results of the biofilm growth at both frequencies in the layers that are 2, 3, 4, and 6 mm thick. The longer the wavelength, the deeper the fluid layer in which the biofilm develops. While in a 3-mm layer at λ ~ 5 mm (*f*_s_ = 120 Hz) no biofilm is formed (normalized biomass is close to one), the biofilm mass doubles in the same layer at λ ~ 10 mm (*f*_s_ = 45 Hz).

The results presented here offer a simple yet efficient method of shaping biofilms on a solid substrate and allow increasing the biofilm development by inducing waves on the surface of the media. In this work, the effect of surface waves on the bacterial attachment and biofilm growth is reported for a model microorganism *E. coli*. Further work needs to be done with other bacterial strains of interest. It would also be interesting to investigate whether waves affect attachment of mammalian cells and the tissue growth. Further studies of the effects of the wave-induced transport on the structure of extracellular matrix of the biofilms and on the structure of bacterial cellulose are under way. Such studies may lead to new ways of sculpturing the cellulose structure for applications in tissue engineering.

## MATERIALS AND METHODS

### Experimental setup

The sample holders housed in a temperature-controlled incubator at 37^o^C are vertically vibrated by an electrodynamic shaker. The vertical acceleration of the microplates is accurately monitored. The frequency of the vertical vibration can be changed in the range of 0 to 1.2 kHz, and the maximum acceleration is up to 20*g*. Most of the results presented in this paper are conducted at 120 Hz, with vertical accelerations in the range from 2 to 7*g*, which corresponds to a vertical displacement of 0.07 mm (2*g*) to 0.24 mm (7*g*). Experiments are also conducted at *f*_s_ = 60 Hz at *a* = 1.5*g* (0.11 mm vertical displacement) to 3*g* (0.4 mm displacement) and at *f*_s_ = 45 Hz at *a* = 0.8*g* (0.1 mm vertical displacement). Polyamid microparticles (0.005 mm) and TiO_2_ particles (0.0004 mm) are used as tracers in the sedimentation studies. The images of the sedimentation are captured using a Andor Zyla camera mounted above the microplates.

### Wave measurement

A synthetic Schlieren technique developed in (*33*) is used to measure wave fields on the water surface. The method is based on the analysis of the refracted image (above the fluid surface) of a random dot pattern placed under the transparent bottom of the fluid plate. When the surface is flat, a reference image is obtained. The apparent displacement field between the refracted image and the reference image is determined, which is then used for the reconstruction of the instantaneous surface elevation measurements. The surface elevation is used to identify and track horizontal motion of oscillons. After preprocessing the images using the ImageJ software, the isolines of the surface elevation are analyzed to identify the oscillons. A particle tracking algorithm is used to follow trajectories of the oscillons using a nearest neighbor algorithm.

### Bacteria culture

Microplates (six-well) containing 2 ml of media [tryptic soy broth + ampicillin (100 μg/ml)] are inoculated with 20 μl of overnight culture of *E. coli* [American Type Culture Collection (ATCC) 25922GFP] diluted to 0.1 OD (at 600 nm). The microplates are vibrated for 24 hours. The samples are further incubated for another 24 hours at 37°C. The control samples are incubated for 48 hours in a nonvibrated incubator. Measurements are performed six times with three repeats each at every vertical acceleration. At the end of each experiment, the top solution from each well of the microplates is collected to measure the OD_600_ to characterize the planktonic bacterial growth. The attached bacteria and biofilms are washed before they are stained using 0.1% CV and incubated for 20 min. After the development of the stain, the samples are washed several times to remove unabsorbed CV. Then, the absorbed CV is dissolved in 2 ml of ethanol solution (20% acetone and 80% ethanol) to measure the OD (at 550 nm) of CV absorbed by the attached biomass. All OD measurements are performed using a Varioskan LUX multimode reader. The 3D structures and movies of biofilms were obtained using an upright Zeiss LSM780 UV-NLO confocal microscope.

To make the inactive bacterial suspension, 2 ml of overnight culture of *E. coli* is centrifuged and the pellet is resuspended in 2 ml of phosphate-buffered saline before the experiment.

## Supplementary Material

aaz9386_Movie_S1.mov

aaz9386_SM.pdf

## SUPPLEMENTARY MATERIALS

Supplementary material for this article is available at http://advances.sciencemag.org/cgi/content/full/6/22/eaaz9386/DC1

## Appendix

View/request a protocol for this paper from *Bio-protocol*.
