# Supplementary material for: Surface waves control bacterial attachment and formation of biofilms in thin layers
Source: Sci Adv. 2020 May 27;6(22):eaaz9386. doi: 10.1126/sciadv.aaz9386 (PMC7385439; doi:10.1126/sciadv.aaz9386)
Supplement: aaz9386_SM.pdf [file aaz9386_SM.pdf]

Science Advances
AAAS

advances.sciencemag.org/cgi/content/full/6/22/eaaz9386/DC1

Supplementary Materials for

Surface waves control bacterial attachment and formation of biofilms in thin layers

Sung-Ha Hong, Jean-Baptiste Gorce, Horst Punzmann, Nicolas Francois, Michael Shats, Hua Xia*

*Corresponding author. Email: hua.xia@anu.edu.au

Published 27 May 2020, *Sci. Adv.* **6**, eaaz9386 (2020)
DOI: 10.1126/sciadv.aaz9386

The PDF file includes:

Figs. S1 to S4
Legend for movie S1

Other Supplementary Material for this manuscript includes the following:

(available at advances.sciencemag.org/cgi/content/full/6/22/eaaz9386/DC1)

Movie S1

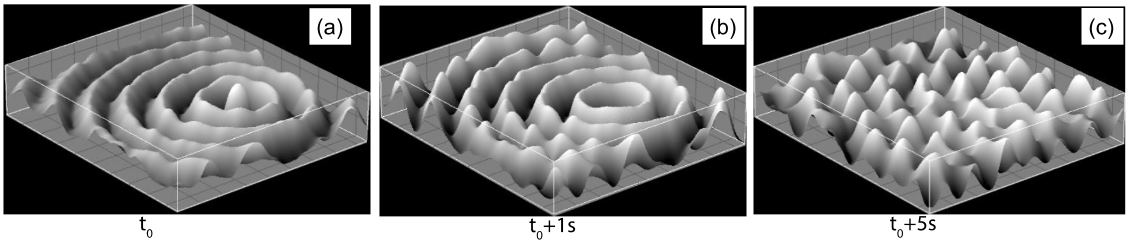

Fig. S1. Development of parametrically excited waves in vertically vibrated containers. The initially regular wave structure, concentric rings in (a), is modulated by the cross-wave instability (b) until the wave becomes broken into a wave field consisting of individual oscillons (c).

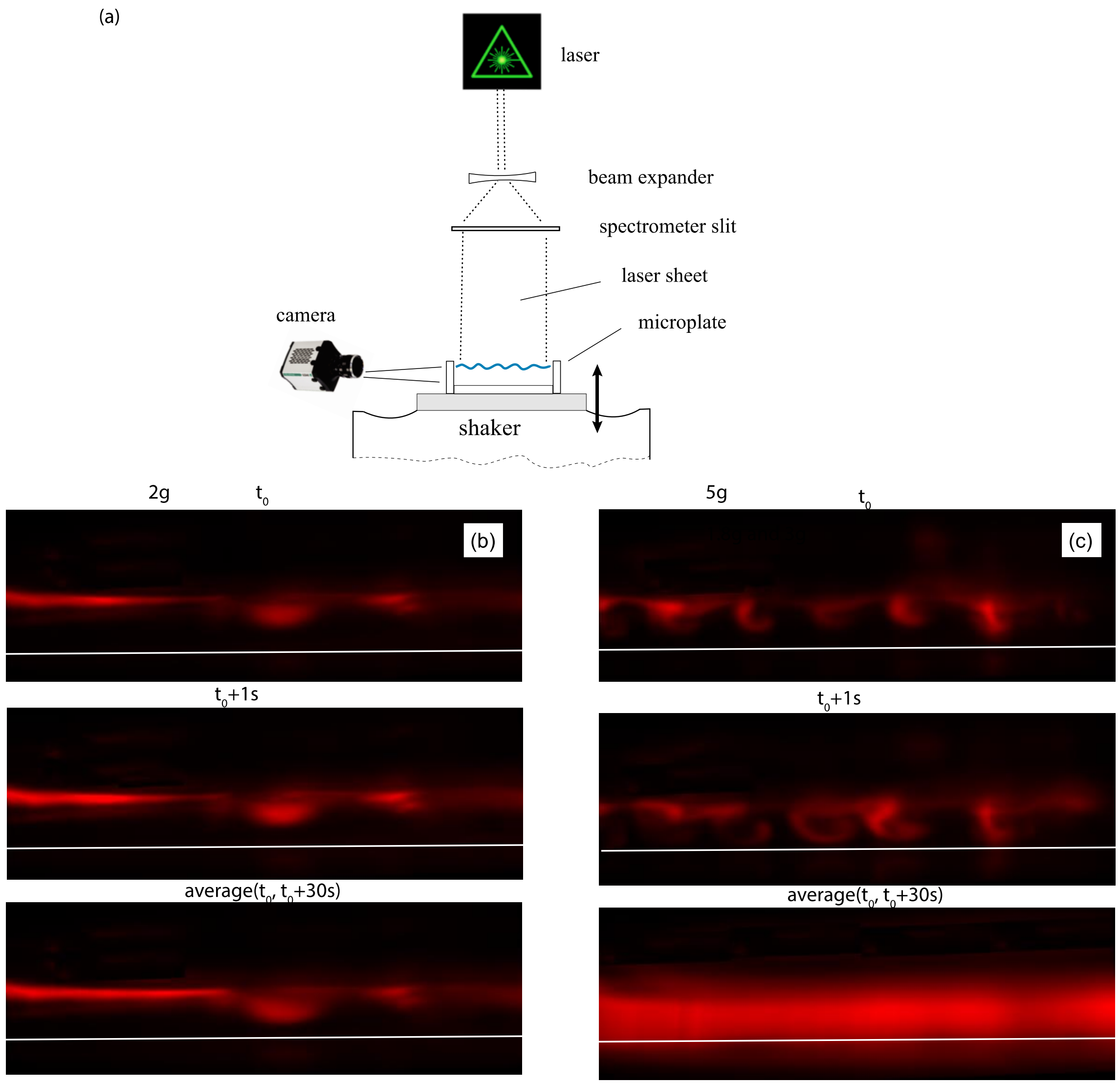

Fig. S2. Visualisation of vertical transport and mixing of a fluorescent dye under the action of the Faraday waves. (a) Experimental setup. A thin (0.1 mm) layer of a fluorescent dye (Rhodamine B) mixed with fresh water is carefully spread on top of saline solution before the Faraday waves are excited. The density gradient leads to a slow molecular diffusion. Without any external agitation, the diffusion of the dye into the fluid becomes noticeable in about 20 min. A vertical (green) laser sheet illuminates the fluid layer in the vertical $z - x$ plane. Initially, only the top layer fluoresces in orange. The fluorescent image is filtered (using an orange filter) before entering the lens of the fast video camera. The dynamics of the dye mixing with the liquid is shown in (b, c) at two instants in time: shortly after the Faraday waves are excited (top row), at t_0, and one second later, at t_0 + 1s (second row). The mixing averaged over 30 seconds is shown in the bottom row. (b) The left column images are obtained at the acceleration of $a = 2g$; (c) the right column images show mixing patterns at $a = 5g$. The white line in each panel shows the bottom of the microplate wells. At lower vertical acceleration ($a = 2g$), vertical plumes of the dye do not substantially move in horizontal direction, while at higher acceleration ($a = 5g$), horizontal mixing within the entire layer is much more intense.

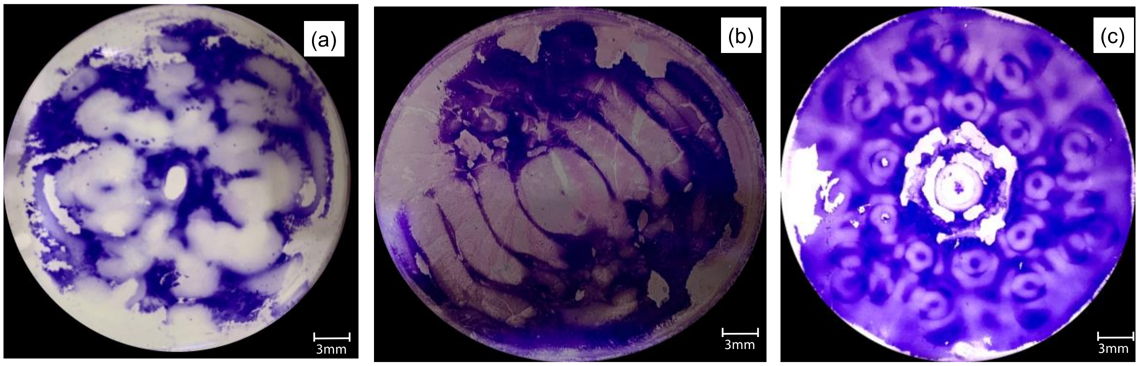

Fig. S3. Images of the biofilms (crystal violet stain) developed at different Faraday wave frequencies. Experimental conditions are: (a) f_s = 45 Hz, vertical acceleration a = 0.8g, (b) f_s = 60 Hz, vertical acceleration of a = 1.5g and (c) f_s = 120 Hz, vertical acceleration a = 3g.

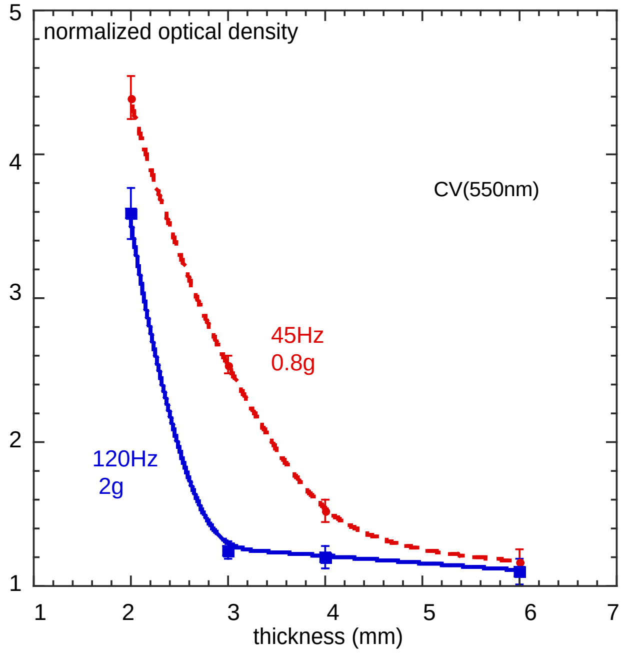

Fig. S4. **Effect of the layer thickness on the biofilm growth at different wave frequencies (wavelengths).** The normalized optical density of the absorbed crystal violet stain at 550 nm for different depths of the layer at (120 Hz, 2g) and (45 Hz, 0.8g) respectively.

Other supplementary files:

Movie S1. Oscillon mobility. Contour plots of the surface elevation are used to identify the oscillons. The video shows that the oscillons chaotically move, collide and merge.
